# Developing a Research Mentorship Program: The American Society of Pediatric Nephrology's Experience

**DOI:** 10.3389/fped.2019.00155

**Published:** 2019-04-24

**Authors:** Tetyana L. Vasylyeva, María E. Díaz-González de Ferris, David S. Hains, Jacqueline Ho, Lyndsay A. Harshman, Kimberly J. Reidy, Tammy M. Brady, Daryl M. Okamura, Dmitry V. Samsonov, Scott E. Wenderfer, Erum A. Hartung

**Affiliations:** ^1^Department of Pediatrics, Texas Tech University Health Sciences Center, Amarillo, TX, United States; ^2^UNC Transition Program, Manning Drive N.C. Children's Hospital, The University of North Carolina, Chapel Hill, NC, United States; ^3^Division of Pediatric Nephrology, Indiana University School of Medicine, Indianapolis, IN, United States; ^4^UPMC Children's Hospital of Pittsburgh, University of Pittsburgh, Pittsburgh, PA, United States; ^5^Division of Pediatric Nephrology, Department of Pediatrics, University of Iowa Stead Family, Iowa City, IA, United States; ^6^Department of Pediatrics, Montefiore Medical Center, Albert Einstein College of Medicine, Children's Hospital at Montefiore, Bronx, NY, United States; ^7^Division of Pediatric Nephrology, Johns Hopkins University School of Medicine, Baltimore, MD, United States; ^8^Center for Developmental Biology and Regenerative Medicine, Seattle Children's Hospital, University of Washington, Seattle, WA, United States; ^9^New York Medical College, Valhalla, NY, United States; ^10^Renal Section, Baylor College of Medicine, Texas Children's Hospital, Houston, TX, United States; ^11^Division of Nephrology, Children's Hospital of Philadelphia, Perelman School of Medicine of the University of Pennsylvania, Philadelphia, PA, United States

**Keywords:** mentorship, research, society, grants, pediatrics

## Abstract

**Background:** Most pediatric nephrologists work in academia. Mentor-mentee relationships provide support and guidance for successful research career. Mentorship program implementation is valuable in medical fields for providing research opportunities to young faculty.

**Methods:** The American Society of Pediatric Nephrology (ASPN) established a research mentorship program to (a) assist with matching of appropriate mentor-mentee dyads and (b) establish metrics for desirable mentor-mentee outcomes with two independent components: (1) the grants review workshop, a short-term program providing mentor feedback on grant proposals, and (2) the longitudinal program, establishing long-term mentor-mentee relationships. Regular surveys of both mentors and mentees were reviewed to evaluate and refine the program.

**Results:** Twelve mentees and 17 mentors participated in the grant review workshop and 19 mentees were matched to mentors in the longitudinal program. A review of NIH RePORTER data indicated that since 2014, 13 NIH grants have been awarded. Mentees in the longitudinal program reported that the program helped most with identifying an outside mentor, improving grant research content, and with general career development. Mentors perceived themselves to be most helpful in assisting with overall career plans. Email communications were preferred over phone or face-to-face communications. Mentees endorsed strong interest in staying in touch with their mentors and 100% of mentors expressed their willingness to serve in the future.

**Conclusion:** This mentorship program was initiated and supported by a relatively small medical society and has shown early success in cultivating mentoring relationships for a future generation of clinician-scientists.

## Introduction

Mentoring is a special partnership between two people based on common goals and expectations, focus, and mutual trust and respect (1). Successful mentoring requires commitment to this process by both mentors and mentees and it serves as an important career advancement mechanism for both (2). Mentors provide guidance and promote a mentee's achievement in any or all of the following: academic pursuits, clinical excellence, life/professional goals and work-life balance. Neely et al. wrote, “Development of an academic career easily follows a clinical course for which there are multiple role models; however, development of an academic research career involves few role models, and rarely do instructional guides reach out to the new faculty” (3). In addition to professional aptitude, two components appear essential for a successful research career: (1) a broad supportive infrastructure, and (2) a person- and time-specific continuous mentor-mentee relationship. Positive mentoring relationships require trust, respect, shared information, resources, and expectations as well as professional, enthusiastic, supportive, and collaborative problem-solving (4).

Although a number of papers and internet resources address mentorship programs (5–8), very few focus on development of an academic research career for physician-scientists (3, 9–12). These resources are mostly institution-based and oriented to students and/or graduate medical trainees (13–15). Two recent papers addressed more sponsorship-type programs instead of structural research mentorships (16, 17). Faculty development programs from medical societies are more oriented toward educational research. For example, the American Society of Hematology developed an educational scholarly project (18) and the American Society of Pediatric Hematology/Oncology supports a mentoring program for early career members (19). The Society for Maternal-Fetal Medicine expressed additional concern about forthcoming training of physician-scientists: uncertainties related to future administration of health care, federal support for research, attrition of physician-scientists, and an inadequate supply of new scholars (20). Furthermore, the Accreditation Council for Graduate Medical Education has increased the scholarly productivity requirements for residency programs, placing even more pressure on faculty to be productive in this realm.

Recognizing the need, the American Society of Nephrology (ASN) recently introduced an extensive set of electronic educational resources, presented in brief animations, podcasts, and other media for internal medicine nephrologists. These resources detail effective communication, identifying the right mentor or mentee, navigating mentorship challenges, and assessing a mentee's understanding of expectations (21). These resources developed by an adult medicine subspecialty professional society addressed universal challenges to academic medicine. Although virtually all ASPN members are also ASN members, gaps remained for those trained in pediatric nephrology.

As with other pediatric subspecialties, many pediatric nephrologists work in small divisions with as few as 1–3 faculty in a group. Thus, the lack of a local, experienced research mentor to guide research development and review early faculty progress is common. Recognizing that many junior investigators could benefit from the addition of established senior investigators to their mentoring team, the ASPN created a research mentorship program in 2014.

The specific goals of the ASPN mentorship program were (1) to provide junior investigators with successful research mentors outside their home institutions who have similar research interests, and (2) to provide mentorship training to more established investigators who are ready to become mentors. This program allowed mentors to support junior faculty career development, academic pursuits, and development of grant funding proposals. In this descriptive paper, we present the unique experiences and early data gathered during the ASPN research mentorship program development and implementation. Our experiences may serve as an example for other medical societies seeking to improve junior faculty research mentoring experiences.

## Methods

### Development of the ASPN Mentorship Program

The ASPN Mentorship Program was conceived and developed by the ASPN Research Committee in 2014 to match mentors with self-identified mentees and establish an environment for desirable mentor-mentee research outcomes. The program was designed to be dynamic and open to modifications based on periodic surveys and evaluations by all participants. In 2016, a Quality Improvement (QI) framework was formalized with creation of an American Board of Pediatrics (ABP)-approved Maintenance of Certification (MOC) Part 4 program. The program was administered by the co-chairs of the Research Committee, who typically serve 3-year terms. ASPN senior leadership (Council of the Society) ensures management support and monitors step-by-step progress reports.

The mentoring program had two components: (a) the short-term grant review program, designed to help a mentee with a specific grant submission and (b) the longitudinal program, which facilitates the establishment of long-term mentor-mentee relationships to aid in career development. Mentors and mentees can choose to participate in one or both components. Furthermore, the mentee can participate in the grant review program as often as desired. One of the most important approaches to ensure success of the program is optimal matching of mentors and mentees.

### Requirements for Mentors and Mentees

The ASPN Research Committee co-chairs selected potential mentors for each mentee among the existing research committee members, current members of the Society, or established non-Society mentors aligned within the field of pediatric nephrology. The selection process was iterative, with feedback from both mentees and potential mentors to ensure no conflicts of interest or conflicts in commitment. To participate in the program, mentees need only to be ASPN members in good standing. ASPN mentees who entered in the longitudinal program perceived that mentors would help with their career and research development.

### Grant Review Program

The grant review program consisted of an annual in-person grant review workshop held in conjunction with a large national meeting. These workshops were held in a “mock study section” format, with 3–4 mentors serving as primary and secondary reviewers for 2–3 grants presented orally by groups of mentees. The structure of these workshops was tailored to the needs of the mentees who were assigned to small groups based on research topic or methodology. Mentors are selected based on proven track record of grantsmanship sufficient for the mentees' stage of training. However, often mid-level and senior investigators were assigned as reviewers for the same career development grants in order to also provide experience for mid-level faculty on grant review. Mentees submitted drafts of their grant applications in advance of the in-person workshop, and the mentors were asked to complete a written review using a standard NIH-style review template. Mentees were required to submit a specific aims page at a minimum and were also encouraged to submit other grant sections (e.g., Research Strategy, Career Development). All participants were asked to keep the review process confidential.

### Longitudinal Program

Mentees and mentors in the longitudinal program signed a Mentoring Partnership Agreement on enrollment to formalize the relationship and clarify individual expectations. These expectations included committing to regular meetings (in person at least annually; by phone or virtually at other time points), maintaining confidentiality, reviewing and revising the mentees' goals statements annually, providing each other with honest, direct, and respectful feedback, and providing program feedback to the Research Committee. Mentees were asked to write a goals statement, to be reviewed/updated annually with their mentors. Mentees were also encouraged to contact the mentor regularly via phone or electronic communication to discuss specific needs related to grant review, programmatic development, promotion, and tenure.

Mentors in the program are experienced, independently-funded physician scientists, who are able to work with mentees to develop goals with realistic expectations and individualized career development plans. Mentors who reviewed grants for mentees also participated in the grant review component were often selected to become a longitudinal mentor. It was important for mentors to commit to meeting routinely with the mentees to review progress, give feedback, provide recommendations for improvements, and guide academic career development (e.g., assist with grant proposals, manuscript submissions, and oral presentations). The Program leadership maintains Mentorship Partnership Agreements but does not review goals statements or drafts of grants/manuscripts, which are only shared between mentees and their mentors.

### Role of the ASPN in Program Promotion and Support

The role of the ASPN in the mentorship program was to advertise the program, elicit participation, judiciously select and contact mentors for each new mentee in the program, review program feedback and outcome metrics regularly to improve the program organization, and develop education workshops. Research Committee members who participated in the QI project had to attend meetings to review survey data at least twice yearly for 2 years to be eligible to receive ABP MOC Part 4 credit.

### Research Mentorship Program Evaluation

Surveys were sent to mentees and mentors using Survey Monkey®. Surveys were designed based on program metrics and questions were left unchanged from year-to-year to allow tracking of program efficacy over time in a standard fashion. Mentees and mentors in the longitudinal program were asked about methods and rates of contact with each other within the context of the program. Mentees and mentors in the grants review workshop were asked about what portions of their grants were specifically reviewed. Metrics selected to rate the quality of the program included expectations, number, and format of meetings between mentors and mentees, type of grants reviewed, number and content of sections of grants reviewed, and perceived helpfulness of mentee-mentor interactions in specific areas (i.e., improving grantsmanship). NIH grants are tracked using the RePORTER database (https://projectreporter.nih.gov/reporter.cfm). Metrics were collated by the ASPN Research Committee co-chairs and reviewed in aggregate at Committee meetings on a quarterly basis. Program improvements were implemented on an ongoing basis.

In response to participants' feedback that the program would benefit from more active oversight from the Research Committee, a mentorship program Oversight Subcommittee was formed in 2017. This Oversight Committee, which consists of former co-chairs of the Research Committee and other senior ASPN members, reaches out to mentees and mentors in the longitudinal program on a regular basis to ensure continued mentor-mentee contact and to provide educational resources.

## Results

### Outcomes of the ASPN Mentorship Program

Since its inception, 12 mentees and 17 mentors have participated in the grant review program and 19 were matched as mentor-mentee pairs for the longitudinal component. Ten mentees participated in both programs, so there have been 21 unique mentees in the mentoring program. Of these, 16 were women and 5 were men. At the time of enrollment in the mentoring program, 17 mentees were junior faculty and 4 were in pediatric nephrology fellowship. Fellowship mentees were also supported by their local Scholarship Oversight Committees. Of the longitudinal program mentees, 8 have “graduated” from their formal mentoring relationship and were able to submit a grant. Mentees were considered to have “graduated” if they had reached their goals within the program, which were delineated at the time of program entry, and therefore chose to formally end their participation. From the 21 mentees who have participated in the mentoring program since 2014, review of NIH RePORTER data in October 2018 indicated 13 successful NIH grants following mentorship program participation: 5 R01s, 3 R03s, and 5 K awards. Although non-NIH funding (commercial, private foundations, institutional internal funds, etc.) represents a substantial source of research support this information was more difficult to track. We are planning to collect this information in our upcoming surveys.

**Figure 1:**
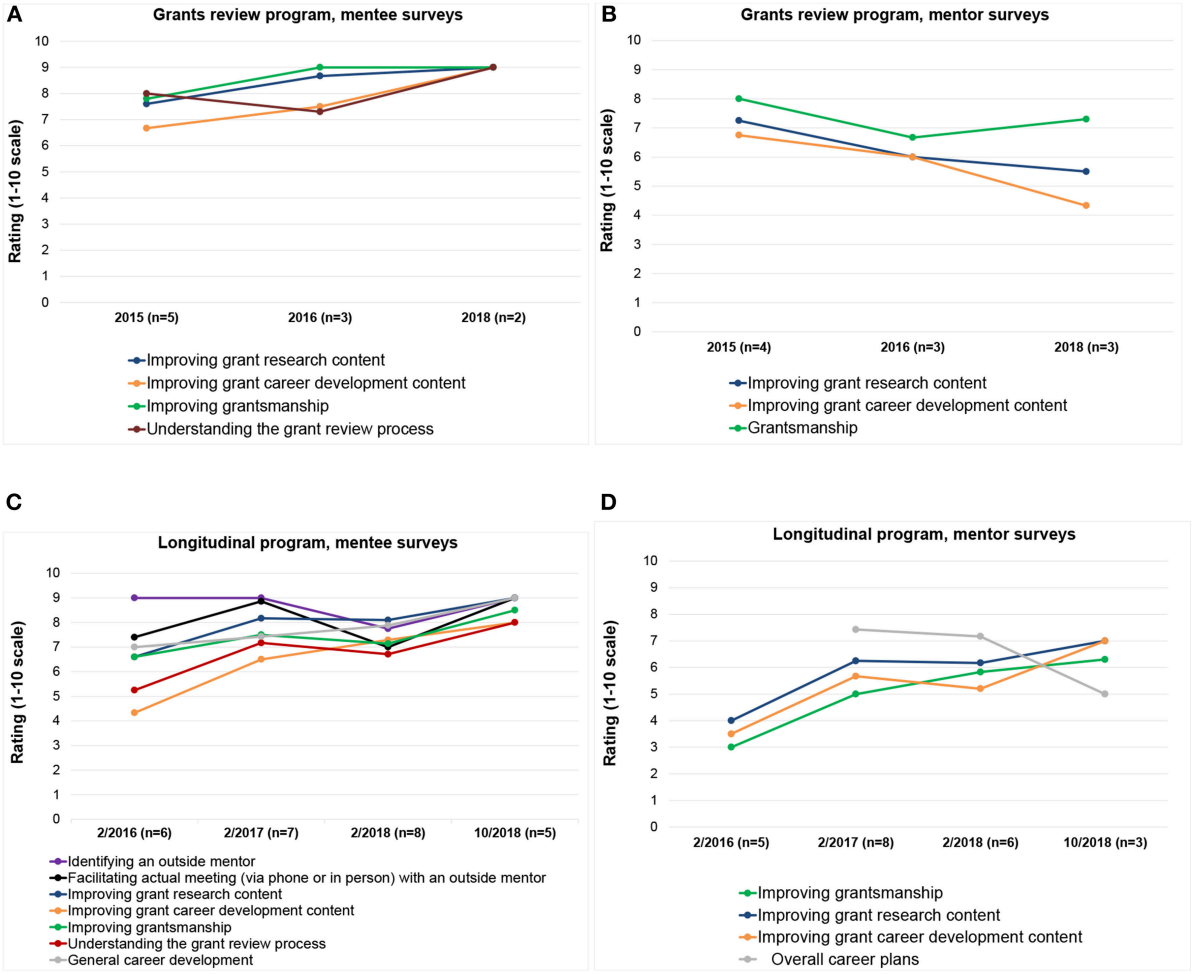
American Society of Pediatric Nephrology research mentorship program: Mentees' ratings of the program's helpfulness in different areas, and mentors' ratings of their own helpfulness to their mentees. **(A)** Grant review mentees, **(B)** Grant review mentors, **(C)** Longitudinal program mentees, and **(D)** Longitudinal program mentors.

Tables 1, 2 detail longitudinal program mentee and mentor responses, respectively, to survey questions regarding expectations, mentee-mentor fit, frequency of communication, future meeting plans. Sections of grant proposals reviewed in the longitudinal program are shown in Table 3. Figure 1 represents satisfaction with the grant review (A) and longitudinal components (B) of the program in various areas.

**Table 1:** American Society of Pediatric Nephrology longitudinal research mentorship program: Mentees' perceptions on expectations and meetings with mentors over a 2-year period, *n* (%) (Questions were added in 2017, so 2016 data is not available).

**Survey year**	**2017** ***n* = 7**	**2018** ***n* = 8**
**Question 1: What were your expectations going into the program?**		
Submit R01	2 (29)	3 (33)
Submit career development grant	4 (57)	5 (56)
Submit institutional or foundation grant	0	0
Formulating a research career	1 (14)	2 (11)
**Question 2: Has this program met your expectations?**		
Yes	6 (86)	8 (100)
No	1 (14)	0
**Question 3: Have you discussed expectations with your mentor?**		
Yes	6 (86)	8 (100)
No	1 (14)	0
**Question 4: In the past 6 months, how many times did you have contact with your mentor?**		
None	2 (29)	1 (12.5)
1	1 (14)	4 (50)
2-3	4 (57)	2 (25)
4 or more	0	1 (12.5)
**Question 5: In what manner did you communicate with your mentor?**		
Email	2 (29)	3 (44)
Phone	3 (43)	2 (29)
In person	2 (29)	(29)
Video chat	0	0
**Question 6: Do you have another meeting scheduled?**		
Yes	1 (14)	4 (50)
No	6 (86)	4 (50)

**Table 2:** American Society of Pediatric Nephrology longitudinal research mentorship program: Mentors' perceptions on expectations and meetings with mentees at the end of the represented year, *n* (%).

**Survey year**	**2016** ***n* = 5**	**2017** ***n* = 8**	**2018** ***n* = 6**
**Question 1: Do you think you were a good fit for your mentee's research focus?**			
Yes	5 (100)	8 (100)	8 (100)
No	0	0	0
**Question 2: In what manner did you communicate with your mentee?**			
Email	5 (100)	8 (100)	6 (100)
Phone	1 (20)	5 (63)	4 (67)
In person	4 (80)	6 (75)	5 (83)
Video chat	1 (20)	0	1 (17)
Question 3: Number of times you met with your mentee?^*^			
None	–	1 (13)	3 (50)
1	–	3 (38)	2 (33)
2–3	–	3 (38)	0
4 or more	–	1 (13)	1 (17)
**Question 4: Would you be willing to serve as a mentor in the future?**			
Yes	4 (80)	8 (100)	6 (100)
No	1 (20)	0	0

*Mentors were surveyed after each year of the active program*.

* *Question added to survey in 2017*.

**Table 3:** American Society of Pediatric Nephrology longitudinal research mentorship program: Mentees' and mentors' reports of which sections of the grant were reviewed [*n* (%) of positive responses].

**Grant sections reviewed**	**Mentees**			**Mentors**		
**Years**	**2016** *n* **= 6**	**2017** *n* **= 7**	**2018** *n* **= 8**	**2016** *n* **= 5**	**2017** *n* **= 8**	**2018** *n* **= 6**
No comment	2 (33)	1 (14)	2 (25)	0	2 (25)	1 (17)
Concept/plan only	1 (17)	1 (14)	3 (38)	4 (80)	2 (25)	4 (67)
Specific aims	3 (50)	5 (71)	6 (75)	1 (20)	6 (75)	3 (50)
Significance	1 (17)	3 (43)	1 (13)	1 (20)	2 (25)	2 (33)
Innovation	1 (17)	4 (57)	1 (13)	1 (20)	2 (25)	1 (17)
Approach	2 (33)	5 (71)	2 (25)	2 (40)	5 (63)	2 (33)
Career development plan	0	1 (14)	1 (13)	0	1 (13)	0

In 2018, 100% of mentees in the longitudinal program agreed that the program met their expectations and that expectations were clearly communicated with the mentors. During a 6 month period, 50% of mentees met with their mentors face to face at least once, 25% met 2–3 times, and 12.5% met 4 or more times (Table 1). The vast majority of the longitudinal program mentees discussed the *Specific Aims* of their grants but interactions were less focused on their overall career development plan (Table 3). For the longitudinal component, email communications were preferred over phone or face-to-face communications.

While every mentor over the initial 3-year-period strongly believed that he/she was a good fit for their mentee, mentors' perceptions of the program differed from mentees' in key aspects. Mentors in both the grants review and longitudinal components of the program perceived their overall helpfulness relatively low on a scale from 1 to 10 (Figures 1B,D), despite the mentees rating the program's helpfulness relatively highly (Figures 1A,C). Although no more than 14% of longitudinal program mentees reported reviewing long term career development plans with their mentors (Table 3), mentees rated the program relatively highly for helpfulness in general career development (score 7.0–7.9 out of 10, Figure 1B). Similarly, mentors also reported that they believed they were helpful to their mentees in “overall career plans” (score 7.2–7.4 out of 10, Figure 1D). As the program has evolved and improved over the years, 100% of mentors expressed their willingness to serve in the future (Table 2). During the course of this program, the longitudinal component demonstrated sustained improvement in satisfaction (Figures 1C,D).

## Discussion

We described the development and management of a junior faculty oriented research mentorship program within a pediatric medical subspecialty. The ASPN mentorship program experience demonstrates participant satisfaction, mentor-mentee stability, programmatic improvement over time, and notably a high yield of NIH grants during the project implementation period. Development of such a program within a relatively small pediatric society is challenging but essential. This model could serve as an example for other similar medical organizations.

The ASPN mentorship program addresses an important gap identified by academic nephrologists at the start of their careers. While some of the challenges experienced by new faculty in pediatric subspecialties are common with other aspiring physician scientists in other medicine careers, some are unique to those in pediatric medicine and nephrology. New faculty in pediatrics can become overwhelmed with the demands of juggling clinical practice with a research career. Initiating research—from conceptualization, proposal development, grant submission and ultimately to implementation and dissemination—is challenging, but extremely important for academic advancement.

The challenges in pediatric nephrology research are many, including a widespread array of pediatric nephrology divisions throughout the country that are small and with a paucity of locally available mentors experienced in multiple areas of investigation (22). In addition, nephrologists have several specific needs that are dependent on patients' unique psychosocial issues and physiology/pathophysiology of kidney disease (23); these issues are only more complicated in children. Several renal-related conditions are relatively uncommon in children, making identification and recruitment of potential clinical study participants extremely difficult.

The small work force in pediatric nephrology complicates the accessibility of young faculty to find research mentorship from established, independently-funded pediatric nephrologists. Through the country, with exception of few large centers, pediatric nephrology programs are represented by 2–5 clinical faculty (24). Moreover, many institutions do not have the infrastructure to support junior/mid-level investigators, which require networking and career guidance from outside mentors. The ASPN mentorship program addressed many of these obstacles by matching experts in the field willing to teach and mentor young investigators even outside their home institutions. Because of modern advances in communication, physical location and distance between mentors and mentees was not taken into consideration, rather their common research interest and desire to pursue the same scientific goals.

### Lessons Learned From the ASPN Mentorship Program

Oversight by the ASPN ensured the success of this program and collection of outcome measurements of the program was critical. Governing the program through the ASPN research committee proved to be a viable option. Customized pairing of mentees with mentors based on academic and research expertise was a high priority for participants. We believe that requesting drafts of mentees' grant applications in advance of the in-person workshop, and requesting that mentors complete a written review, substantially increased the success rate of the program because it ensured that participants were highly motivated.

Mentees highly regarded the ability to receive individualized feedback from established investigators on their research grant proposals. Although not employed by ASPN, similar programs might use an “intelligent match” to achieve the best possible arrangements, using a very specific matching parameters and computerized approach. E-mail was the preferred mode of communication to foster ongoing relationship, with mentors further appreciating the importance of additional face-to-face contact.

We learned that the design and maintenance of a mentoring program should be dynamic with frequent assessments for improvement. An efficient monitoring vehicle was brief periodic online surveys. The frequency of these surveys could depend on the program, but annual surveys were adequate in this experience. In our program response to survey was close to 50% of participants. Results of the surveys were reviewed and discussed both, at meetings and conference calls with the Research Committee to identify process improvements. As a testament to the success of this program, 50% of the mentees surveyed in 2018 had already scheduled another meeting with their mentors, and all mentors were willing to continue sharing their expertise. We also believe that this program helped to enrich a national network of pediatrician scientists with similar research interests. It is possible that people with positive experiences were more likely to complete the survey. As such, some results may be biased toward positive experiences.

Challenges for a mentoring program in academic pediatric nephrology are great but not insurmountable. Limited funding opportunities across a small number of funding agencies in the field can make it difficult for junior investigators to identify and successfully compete for grant funding. Within small programs, mentees may have difficulty finding a mentor with the same or similar research interest or appropriate expertise. Mentors may have topical expertise but lack experience in pediatric research or grant review. Faculty from smaller programs may not have access to robust grant writing or career development curricula. Harnessing the strengths of pediatric academic societies addresses these challenges by bringing together mentees and outside mentors from different institutions.

We were proud of the fact that a significant number of early and midcareer women scientists benefited from the program. Per national NIH data, women are still under-represented at every stage of academic advancement. In 2015, for example, women were 44% of assistant professors, and 35% of professors (25). Our program successfully overcame that disparity.

Our program has some limitations. Due to the relatively small number of participants, we cannot provide detailed characteristics on age, race/ethnicity, and size/resources of their home institution. Although we chose mentors with strong track records of NIH-funded research, we did not collect information about their mentoring activities within their own institutions. In the future we are planning to collect more data about mentee's institutional environment (e.g., size of practice, characteristics of medical centers, local mentoring) as well as data about number of proposals funded by the NIH, industry and foundations.

### Suggestions for the Design of Mentoring Programs

It is fundamental that mentors and mentees who entered a mentoring program have a clear understanding of the goals, objectives, and desirable outcomes of the collaboration. Mentors should be willing to commit to working one-on-one with mentees and ideally should have a successful track record of grant awards and research publications. Mentors should help young clinical scientists to plan, develop, grow, and manage their careers. Mentors also have an important role in helping junior faculty members become resilient in times of change, more self-reliant in their careers, and more responsible as self-directed learners. Mentees should clearly understand expectations and should be willing to provide feedback to the program.

Based on our ASPN experience, we identified that the 10 key elements (in no specific order) for successful mentorship programs are to:

1. Provide concrete advice on specific research pursuits and promote interaction between mentors and mentees as well as other content experts with successful research careers;
2. Provide infrastructure that enables pairing of mentors and mentees, completion of goals statements, assurance of confidentiality, and successful program completion;
3. Facilitate planning and management of program assessments, track scheduling, and report documentation;
4. Inform program participants about available specialty focused NIH and non-NIH funding opportunities;
5. Complete thorough and consistent evaluations of the participants' progress;
6. Perform continuous review and evaluation of program assignments and activities throughout the program cycles and make appropriate changes;
7. Employ continuous QI techniques (Plan-Do-Study-Act) to improve programmatic effectiveness;
8. Provide enticements for senior investigators and experienced mentors to stay involved, such as Maintenance of Certification (MOC) Part 4 credits from the American Board of Pediatrics (ABP) for participants;
9. Train a future generation of mentors through role modeling and feedback.
10. Motivate others with dedication, enthusiasm and good will.

In summary, a structured and well-organized research mentorship program with strong medical society leadership, brings professional fulfillment to academic physicians. In addition, providing increased research mentorship helps to increase and sustain the research workforce, which results in more robust research and improved child health.

## Conclusion

The ASPN Research Mentorship Program, initiated and supported within a relatively small medical society; showed early success in training a future generation of clinician-scientists who will be able to undertake valuable research that we hope will 1 day lead to better patient outcomes.

## Author Contributions

TV participated in the program and led the writing group, correspondent author. MD-G substantial contribution in Program improvement and correction of manuscript. DH led a mentorship program, participated in writing. JH led mentorship program, participated in writing. LH actively participated in mentorship program and manuscript writing. KR actively participated in mentorship program evaluation and manuscript writing. TB led the program, writing. DO led the program, writing. DS manuscript revision, suggestions, writing. SW led the program, participated in survey, data analysis and writing. EH led the program, survey, data analysis, graphs for paper, writing and revisions.

### Conflict of Interest Statement

The authors declare that the research was conducted in the absence of any commercial or financial relationships that could be construed as a potential conflict of interest. The handling Editor declared a shared affiliation at the time of review, though no other collaboration, with one of the authors JH.
